# Correction: Russo et al. The Role of Dielectrophoresis for Cancer Diagnosis and Prognosis. *Cancers* 2022, *14*, 198

**DOI:** 10.3390/cancers16193258

**Published:** 2024-09-25

**Authors:** Giorgio Ivan Russo, Nicolò Musso, Alessandra Romano, Giuseppe Caruso, Salvatore Petralia, Luca Lanzanò, Giuseppe Broggi, Massimo Camarda

**Affiliations:** 1 Urology Section, University of Catania, 95125 Catania, Italy; 2 Department of Biomedical and Biotechnological Science (BIOMETEC), University of Catania, 95123 Catania, Italy; 3 STLab s.r.l., Via Anapo 53, 95126 Catania, Italy; massimo.camarda@stlab.eu; 4 Haematological Section, University of Catania, 95125 Catania, Italy; sandrinaromano@gmail.com; 5 Department of Drug and Health Sciences, University of Catania, 95125 Catania, Italy; forgiuseppecaruso@gmail.com (G.C.); salvatore.petralia@unict.it (S.P.); 6 Department of Physics and Astronomy “Ettore Majorana”, University of Catania, 95123 Catania, Italy; luca.lanzano@dfa.unict.it; 7 Pathology Section, Department of Medical, Surgical Sciences and Advanced Technologies “G.F. Ingrassia”, University of Catania, 95123 Catania, Italy; giuseppe.broggi@gmail.com

Reference

In the original publication [1], there was a mistake in the legend for Figure 1; The wrong reference was cited. A correction has been made to ref [14]; the updated reference is as follows:

14. Pesch, G. On the Dielectrophoretic Particle Retention in Porous Media. Available online: http://nbn-resolving.de/urn:nbn:de:gbv:46-00106404-11 (accessed on 6 December 2021).

The authors apologise for any inconvenience caused and state that the scientific conclusions are unaffected. This correction was approved by the Academic Editor. The original publication has also been updated.
